# Geological Survey of the State of Tennessee

**Published:** 1833

**Authors:** 


### IV. Geological Survey of the State of Tennessee.

The State of Tennessee has employed professor Troost, of the University of Nashville, to make a geological and mineralogical survey of that state; an enterprize in which he is at this time actually engaged. Every sister state in the West, should follow the example; and every physician should use his influence to promote an object of such deep interest to the profession. Diversities in soil are prolific sources of diversities in disease; and inquiries into the remote causes of cholera morbus, dysentery, autumnal fever, goitre, calculus, and many other maladies, cannot be prosecuted on scientific principles, without a general knowledge of geology and mineralogy. Every physician should have this, and, from personal observation, be able to connect with the history which he may attempt to give of the endemic diseases of the region in which he practises, a scientific account of its structure and mineralogical productions.

